# Use of tobacco and other illicit drugs among adolescent boys and young men in Kampala, Uganda: A result of low parental attention?

**DOI:** 10.1371/journal.pone.0297163

**Published:** 2024-03-26

**Authors:** Alex Mulyowa, Tonny Ssekamatte, Steven N. Kabwama, John Ssenkusu, Rhoda K. Wanyenze, Joseph K. B. Matovu

**Affiliations:** 1 Department of Disease Control and Environmental Health, Makerere University School of Public Health, Kampala, Uganda; 2 Department of Epidemiology and Biostatistics, Makerere University School of Public Health, Kampala, Uganda; 3 Busitema University Faculty of Health Sciences, Department of Community and Public Health, Mbale, Uganda; University of Connecticut Health Center: UConn Health, UNITED STATES

## Abstract

**Introduction:**

Although the use of illicit drugs is common among young people, limited data exists on the use of illicit drugs among adolescent boys and young men (ABYM). We assessed the use of tobacco, marijuana and khat among ABYM to inform the design of harm-reduction interventions.

**Methods:**

This secondary analysis uses data from a formative study conducted among 2,500 ABYM across the five divisions of Kampala between July and August 2020. Survey questions were adopted from the Global Youth Tobacco Survey and Global School-based Student Health Survey. We computed proportions of ABYM that had ever used any form of tobacco or marijuana or khat in the past year and the proportion that had used any of these products in the past 30 days (i.e. ‘current users’). In a sub-group analysis, we assessed if any patterns existed between ABYM’s use of illicit drugs and selected parental attention attributes.

**Results:**

Of 2,500 ABYM, 47.3% (n = 1,182) were aged 15–19 years. Overall, 16.4% (n = 410) reported past-year use of tobacco products while 11.6% (n = 289) and 10.5% (n = 263) reported past-year use of marijuana and khat, respectively. Current use of any illicit drugs was higher among past-year khat (46%, n = 133) and marijuana users (36.9%, n = 97) than past-year tobacco users (4%, n = 17). Current use of any illicit drugs was higher among out-of-school than in-school ABYM and increased with increasing age and education levels. However, parental attention was lower among out-of-school than in-school ABYM and decreased with increasing age and education levels.

**Conclusion:**

Use of illicit drugs is common among ABYM and increased with age and education levels but parental attention among current illicit drug users decreased with increasing age and education levels. These findings suggest that interventions intended to improve parental attention among illicit drug users may help to reduce the prevalence of illicit drug use among ABYM.

## Introduction

The prevalence of drug use is increasingly higher in the current compared to the past generations [1]. Adolescence and young adulthood is characterized with an increase in the use of illicit drugs and other substances, including tobacco [2]. The adolescence period of 10–17 years is a critical risk period for the initiation of substance use, which often peaks at 18–24 years [3]. For tobacco use alone, global trends between 1999–2018 showed that it averaged at 18% among school-going adolescent boys as compared to 11.5% among girls [4]. Adolescent boys who initiate tobacco use at an early age are also prone to coexisting behaviors such as use of marijuana and khat [5–7].

There exists a contextual interplay of geographical location in influencing tobacco and illicit drug use behavior among young people [8]. Urban settings for example, are typically characterized by rampant illicit drug dealing and use [9]. Specific constructs of urban living, such as greater acceptance of, access to, availability of and tolerance to drugs increase predisposition to tobacco and illicit drug use behaviors [10–12]. In Uganda, adolescent boys and young men (ABYM) form a significant proportion of the urban dwellers [13]. They are faced with urban challenges such as easy accessibility to illicit products, social media influences, and adverts promoting risky drug use behavior. These challenges have potential to lead to poor health outcomes for ABYM.

Several factors influence ABYM’s use of tobacco and illicit drugs. The desire to experiment, peers that smoke, recurrent advertisements, and lack of parental supervision influence the onset and continuation of smoking among adolescents [14–16]. Likewise, adolescents’ family networks highly influence their substance use behavior [17]. For example, Baumrind’s [18] parenting style theory suggests that there is a close relationship between parenting style and behavior of adolescents. This is supported by evidence from elsewhere [19–21]. The psychosocial patterns of behavior due to use of tobacco, marijuana or khat thus remain a subject of debate [22,23].

Research on the influence of parental attention on illicit drug use among adolescent boys and young men within the Ugandan context is largely limited. However, although evidence suggests that challenges due to illicit drug use, such as risky sexual behavior and suicidal ideation are greater among males than females [24,25], the present discourse of interventions rarely targets boys. Besides, recent studies have only focused on in-school adolescents [26], but data for the very young adolescents (10–14 years) are usually missing, since most surveys enroll participants aged 15 years or older. To address these gaps, we assessed the use of tobacco and other illicit drugs among ABYM aged 10–24 years in Kampala, Uganda, to inform the design of appropriate interventions to reduce the prevalence of these illicit drugs among ABYM.

## Methods

### Study site

The study was conducted among ABYM within the five divisions of Kampala, the Capital City of Uganda. The five divisions of Kampala are Central, Makindye, Kawempe, Nakawa and Rubaga. Kampala is one of the most populated districts in Uganda, with a population of about 1,650,800 people, among whom 781,700 are males [27]. Of these, 17.6% (137,414) are young men aged below 24 years of age [28]. Most young men are involved in trade, transport (*Boda Boda* cyclists), hawking and automobile repair. Kampala City was chosen as the study site because of its growing young urban population and the likely presence of a high proportion of young people engaging in illicit drug use as has been documented in previous studies [29,30]. This study focused on adolescent boys and young men because of the need to fill a knowledge gap in terms of what is happening with regards to adolescent boys and young men’s health status, so as to generate data necessary to inform the design of interventions targeting this group.

### Study design

The study from which the data analyzed for this paper was a cross-sectional study conducted among 2,500 in-and out-of-school adolescent boys and young men aged 10–24 years, residing in the 5 divisions of Kampala, between July and August 2020 [31]. The total sample population from the main study was used in this secondary analysis.

### Sampling and sample size determination

The detailed sample size calculations and sample size determination have been described elsewhere [31]. Briefly, assuming a type-1 error of 5%, p = 0.14 (proportion of adolescent boys that have ever used drugs in Kampala, Uganda [30]; a design effect of 2.0; a margin of error of 0.05, five divisions in Kampala and a non-response rate of 0.10, we obtained a minimum sample of 2,060 ABYM. This sample size was adjusted to 2,500 to increase the power of the study. The study used multistage sampling to select the study respondents. Initially, 50% of wards/parishes in each division were purposively selected. Then, 20% of the villages in each parish were selected using a random number generator. Within each village, the number of households to be included in the survey was determined using sampling proportionate to size. ABYM aged 10–24 years were enrolled into the study at household level. Both in- and out-of-school ABYM were interviewed, with the sample size allocation for each division distributed using percentages of children 6–12 years not in school, obtained from the Uganda National Population and Housing Census report [32] as a proxy. This resulted into 1,869 in-school and 631 out-of-school ABYM.

For in-school ABYM, one eligible ABYM (i.e. ABYM who was in school within the respective city division) was interviewed from each selected household, until the required number of in-school ABYM was attained. Although the intention had been to conduct interviews for in-school ABYM at the respective schools where they went, this was not possible because the study was conducted when the schools were still under the COVID-19 lockdown. Thus, in-school ABYM were selected on the basis of their schooling at a school (with a known location) within the city division of enrolment. Where applicable, this information was verified with a physical examination of school records from the enrolled ABYM. For out-of-school ABYM, since there was no sampling frame, the Lot Quality Assurance Sampling (LQAS) methodology [33,34] was used to enroll a minimum of 19 respondents per city division from their areas of location or occupation such as garages, *Boda Boda* taxi motorcycle stages, mobile traders and construction sites. The assumption was that it would be easier to recruit out-of-school ABYM at those sites than reaching them through their households, based on our prior knowledge of their working patterns. This was done until the required number of out-of-school ABYM was attained. It is important to note that wherever a non-response was gotten, an effort was made to replace that participant. Therefore, an ABYM who refused to participate or who was not found at home was replaced until the sample size of 2500 was attained. The response rate was thus 100%.

### Data collection procedures and methods

Data were collected through face-to-face interviews. The Global School-based Student Health Survey (GSHS) tool [35], a WHO validated self-administered questionnaire designed to capture data on students aged 13–17 years, was adopted. The questionnaire was expanded to collect data from out-of-school ABYM, aged 20–24 years, and modified into an interview-administered questionnaire. Tobacco use questions were adopted from the Global Youth Tobacco Survey [36]. Questions on use of marijuana and khat were adopted from the GSHS tool. The interviewer-administered questionnaire (S2 File) was pilot-tested, and then uploaded on mobile phones enabled with *KoboCollect*, an android data collection application. Data were collected on use of tobacco, marijuana and khat within the past 12 months and past 30 days preceding the interview; parental attention to ABYM in general and parental attention to ABYM currently using tobacco, marijuana or khat. Twenty-five interviewers were used to collect data in the five Divisions of Kampala City over a two-month period–but these were distributed across each Division based on the allocated number of interviews in that Division. Teams worked Monday to Saturday of each week, using Sunday to rest and refresh for the new week. With six days per week, each team had up to 48 days to complete data collection, including time to locate respondents as well as move from one Ward/Cell or school to another.

### Measurement of variables

Use of tobacco (coded as Yes = 1 and No = 0) was determined using the questions; *Have you ever tried or experimented with any form of smoked tobacco products; Have you ever tried or experimented with smokeless tobacco products*? Responses were summed up to generate a composite variable; ‘Any tobacco use in the past 12 months’. If respondents indicated that they had ever used any form of tobacco products, they were asked if they had ever tried to stop using tobacco, and if they think that they had the ability to stop using tobacco. Use of marijuana or khat (coded as Yes = 1 and No = 0) were determined using the questions: *During the past 12 months*, *how many times have you used marijuana; during the past 12 months*, *how many times have you used khat*, respectively. We then generated a composite variable; ‘use of marijuana and khat in the past 12 months’. ABYM who reported that they used any of the illicit drugs in the past year were asked about use of these drugs in the past 30 days, hereafter designated as “current users”. Current illicit drug users were asked about how often (*always*, *sometimes*, *never*) they received parental attention based on a pre-determined set of parental attention attributes (e.g. being comforted by parents or guardians, being understood by parents or guardians and being given attention and listened to by parents or guardians). We computed the proportion of illicit drug users who reported parental attention in response to a specific set of questions, e.g. *During the past 30 days*, *how often did your parents or guardians comfort you; or during the past 30 days*, *how often did your parents or guardians understand you*? Each question was coded 1 = Always, 2 = Sometimes or 3 = Never. Only current illicit drug users who reported that they always received parental attention were considered as receiving the adequate level of parental attention that they deserve.

### Data analysis

We computed descriptive statistics to describe ABYM’s sociodemographic characteristics, prevalence of tobacco, marijuana or khat use, smoking cessation among current tobacco users, and parental attention towards ABYM using tobacco, marijuana or khat in the past 30 days. Data are presented as frequencies and percentages. Data analysis was done using STATA version 14.

### Ethical considerations

The study protocol was approved by the Higher Degrees, Research and Ethics Committee of Makerere University School of Public Health (Protocol number: 757) and the Uganda National Council for Science and Technology (Study number: 5240). Written informed consent was sought from each of the study participants prior to their participation in the interviews. Parental consent was waived for emancipated minors that were married, had a child or catered for their own livelihood, as defined in the guidelines by the Uganda National Council for Science and Technology [37]. Consent from a parent/guardian was sought for participants in school and below 18 years of age, as well as those out of school but under the care of a parent/guardian. Participants aged 10–17 years were also asked to provide their own assent in addition to the parental consent form. No individual identifying information was collected on the respondents.

## Results

### Respondents’ characteristics

Table 1 shows the sociodemographic characteristics of the respondents. Of the 2,500 ABYM, 74.8% (n = 1869) were in-school; 19.3% (n = 483) were aged 10–14 years, while 22.5% (n = 421) were in primary school.

**Table 1:** Socio-demographic characteristics of adolescent boys and young men in Kampala, Uganda.

Variable	Frequency	Percentage
**Schooling status**		
In-school	1869	74.8
Out-of-school	631	25.2
**Age-group**		
10–14	483	19.3
15–19	1182	47.3
20–24	835	33.4
**Education level**		
Primary	421	22.5
Lower secondary	826	44.2
Upper secondary	397	21.2
Tertiary	225	22.0
**Division**		
Central	257	10.3
Kawempe	617	24.7
Makindye	405	16.2
Nakawa	720	28.8
Rubaga	501	20.0

### Tobacco use among adolescent boys and young men

Table 2 shows the proportion of ABYM who used any form of tobacco products in the past year preceding the interview. Overall, 16.4% (n = 410) used any tobacco product in the past year. Past-year use of any tobacco product was higher among out-of-school ABYM (30.4%, n = 192) than those who were in-school, and increased with age and education levels. By City Division, use of any tobacco products was higher in Kawempe Division (22.5%, n = 139) than in Nakawa Division (9.6%, n = 69).

**Table 2:** Past-year use of any tobacco products among adolescent boys and young men in Kampala by background characteristics.

Background characteristics	Total (N)	Use of any tobacco product in the past year		
		Smoked tobacco products (n, %)	Smokeless tobacco products (n, %)	Any tobacco use in the past 12 months (n, %)
**Overall**	2500	386 (15.4)	83 (3.3)	410 (16.4)
**Schooling status**				
In-school	1869	205 (11.0)	28 (1.5)	218 (11.7)
Out-of-school	631	181 (28.7)	55(8.7)	192 (30.4)
**Age-group**				
10–14	483	14 (2.9)	0 (0.0)	14 (2.9)
15–19	1182	132 (11.2)	29 (2.5)	141 (11.9)
20–24	835	240 (28.7)	54 (6.5)	255 (30.5)
**Education level**				
Primary	421	14 (3.3)	1 (0.2)	14 (3.3)
Lower secondary	826	73 (8.8)	11 (1.3)	78 (9.4)
Upper secondary	397	57 (14.4)	8 (2.0)	61 (15.4)
Tertiary	225	61 (27.1)	8 (3.6)	65 (28.9)
**Division**				
Central	257	40 (15.6)	7 (2.7)	42 (16.3)
Kawempe	617	132 (21.4)	33 (5.4)	139 (22.5)
Makindye	405	67 (16.5)	15 (3.7)	73 (18.0)
Nakawa	720	66 (9.2)	11 (1.5)	69 (9.6)
Rubaga	501	81 (16.2)	17 (3.4)	87 (17.4)

Table 3 shows the proportion of ABYM who reported smoking cessation among adolescent boys and young men who used tobacco in the past year. Nearly 7% (n = 27) of past-year tobacco users had ever tried to stop tobacco use. We found that having the ability to stop tobacco use, among those who had ever tried to stop, was more common among ABYM that were out-of-school (80.9%, n = 17); those aged 20–24 (90.0%, n = 18); and ABYM living in Kawempe (90.9%, n = 10).

**Table 3:** Smoking cessation among adolescent boys and young men who used tobacco in the past 12 months, by background characteristics.

Background characteristics	Any tobacco use in the past year (N)	Ever tried to stop tobacco use (n, %)	Ability to stop tobacco use, among those who have ever tried to stop tobacco use (n, %)	Any tobacco use in the past 30 days (n, %)
**Overall**	410	27 (6.6)	21 (77.8)	17 (4.1)
**Schooling status**				
In-school	218	6 (2.6)	4 (66.7)	2 (0.9)
Out-of-school	192	21 (10.9)	17 (80.9)	15 (7.8)
**Age-group**				
10–14	14	0 (0.0)	0 (0.0)	0 (0.0)
15–19	141	7 (5.0)	3 (42.9)	5 (3.5)
20–24	255	20 (7.8)	18 (90.0)	12 (4.7)
**Education level**				
Primary	14	0 (0.0)	0 (0.0)	0 (0.0)
Lower secondary	78	3 (3.8)	2 (66.7)	0 (0.0)
Upper secondary	61	1 (1.6)	0 (0.0)	1 (1.6)
Tertiary	65	2 (3.1)	2 (100.0)	1 (1.5)
**Division**				
Central	42	2 (4.8)	1 (50.0)	1 (2.4)
Kawempe	139	11 (7.9)	10 (90.9)	8 (5.8)
Makindye	73	4 (5.5)	3 (75.0)	4 (5.5)
Nakawa	69	6 (8.7)	5 (83.3)	0 (0.0)
Rubaga	87	4 (4.6)	2 (50.0)	4 (4.6)

Table 3 further shows the proportion of ABYM that reported any tobacco use in the past 30 days, among past-year users. Of these, only 4% (n = 17) reported that they had used any form of tobacco products in the 30 days preceding the interview. Tobacco use within the past 30 days was higher among out-of-school ABYM (7.8%, n = 15). Past 30-day use of any form of tobacco products increased with increasing age; from 3.5% (n = 5) among ABYM aged 15–19 years to five percent (n = 12) among those aged 20–24 years.

#### Use of marijuana or khat among adolescent boys and young men

**Fig 1:**
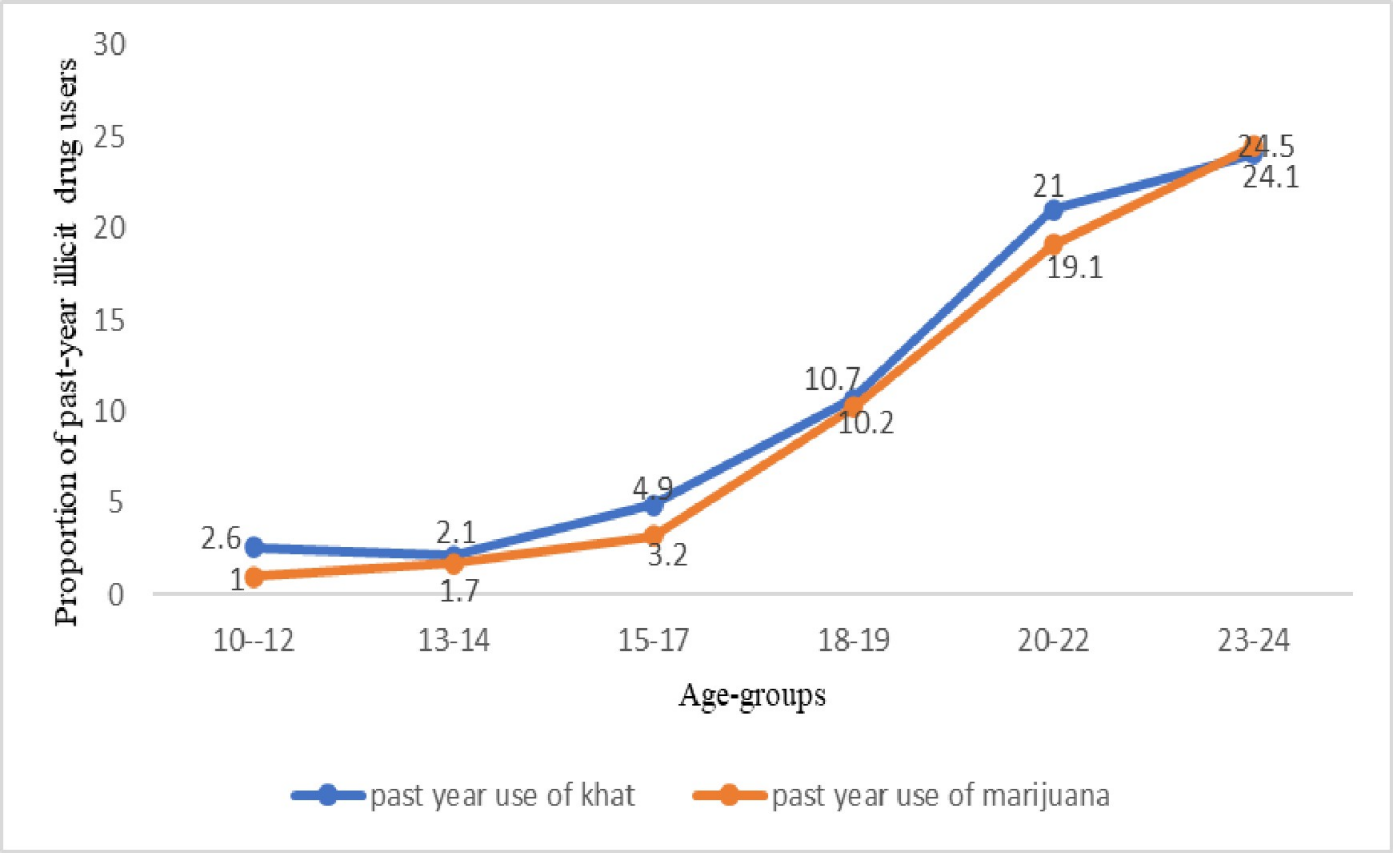
Past-year khat and marijuana use across age-group.

**Fig 2:**
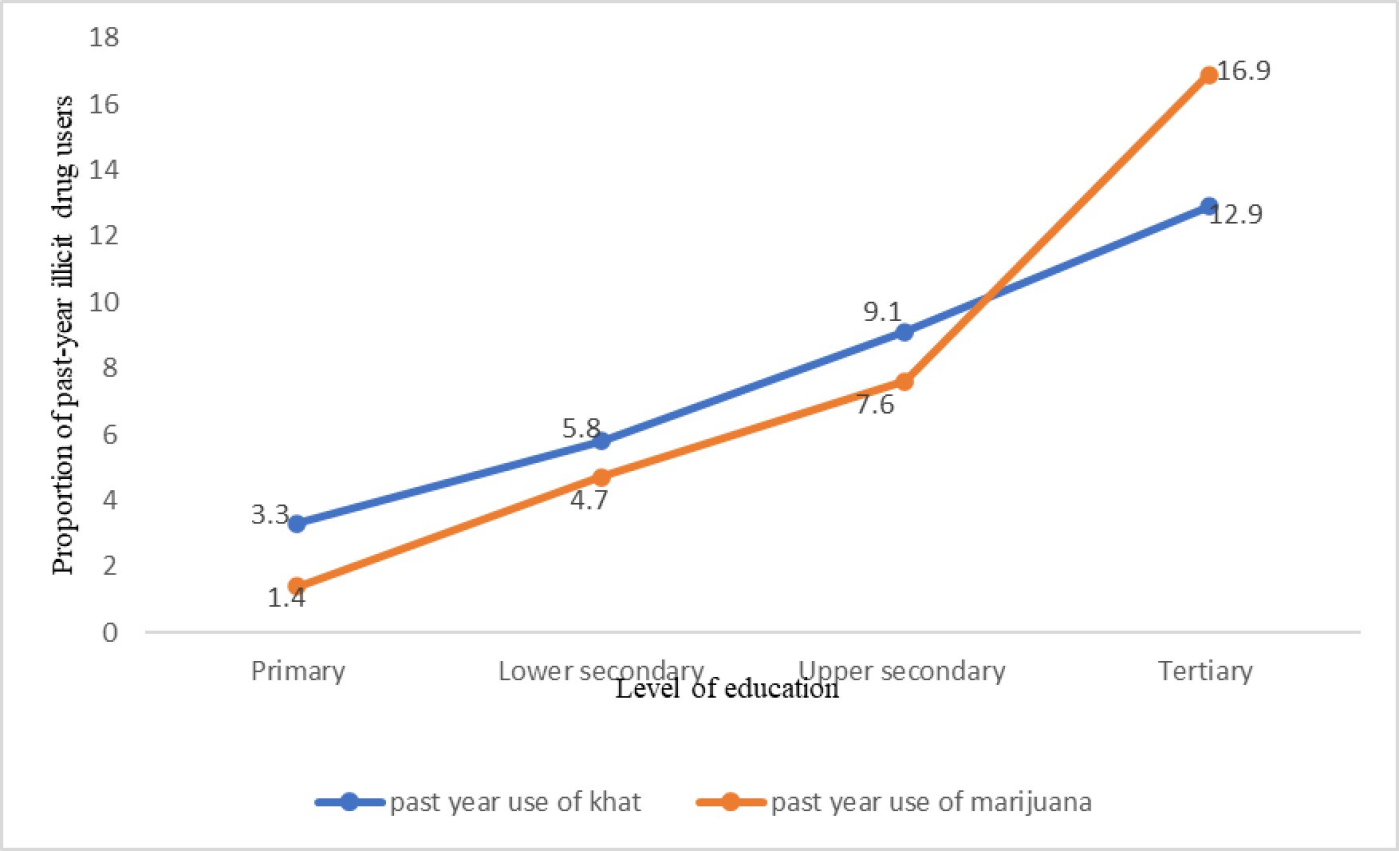
Past-year khat and marijuana use across level of education.

Overall, past-year use of either drug was 11.1% (n = 390). Past-year use of either drug was higher among out-of-school than in-school ABYM (33.8%, n = 213 *vs*. 9.5%, n = 177). In sub-group age stratification, we found that past-year use of either drug increased with increasing age (**Fig 1**) and level of education (**Fig 2**). Important to note is that use of any illicit drug is very low between the ages of 10–12 and 13–14 years but sharply increases from age 15 to age 24. Similarly, past-year use of any illicit drug is very low among those with primary education but sharply increases among those with secondary and tertiary education.

**Fig 3:**
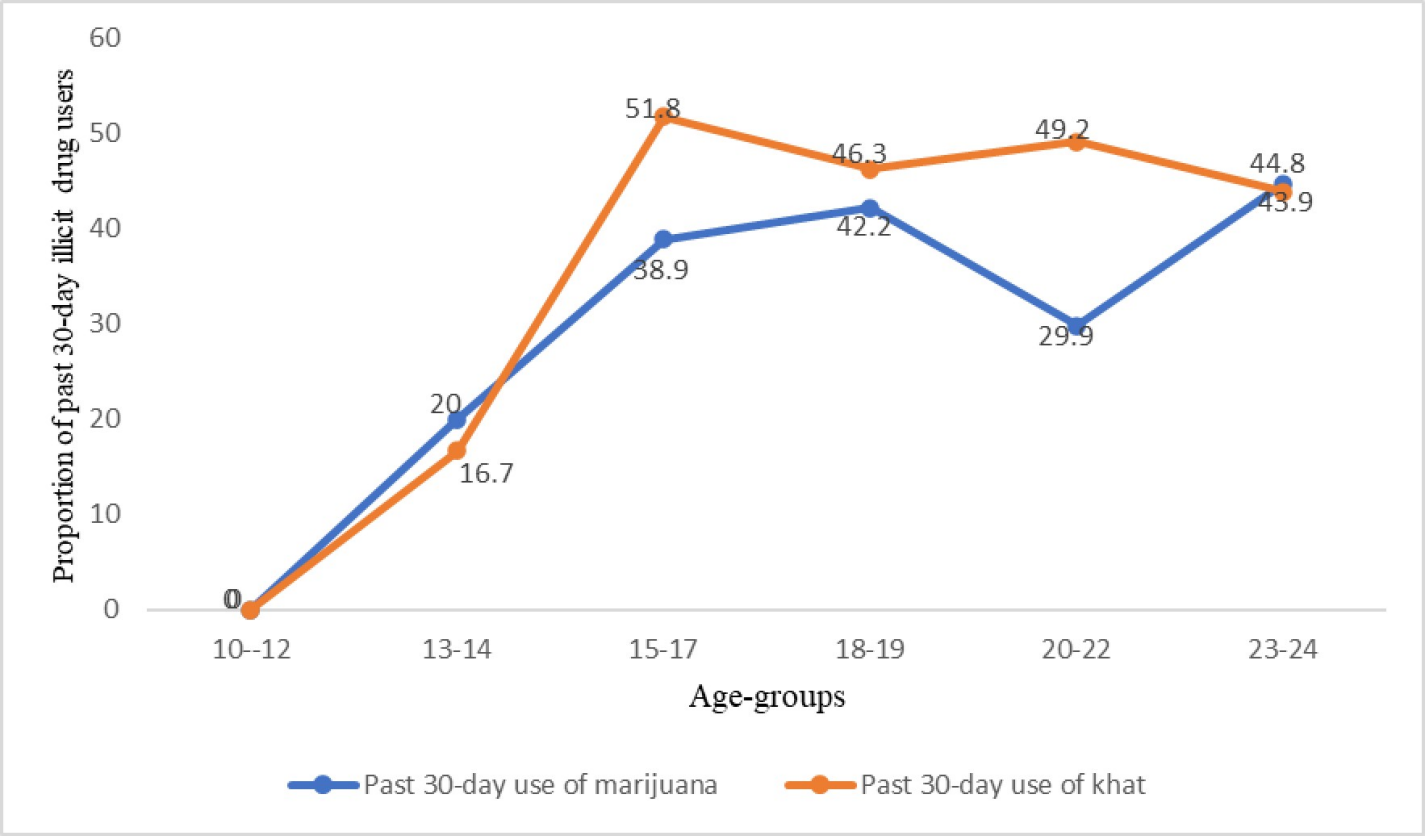
Past 30-day marijuana and khat use across age-group.

**Fig 4:**
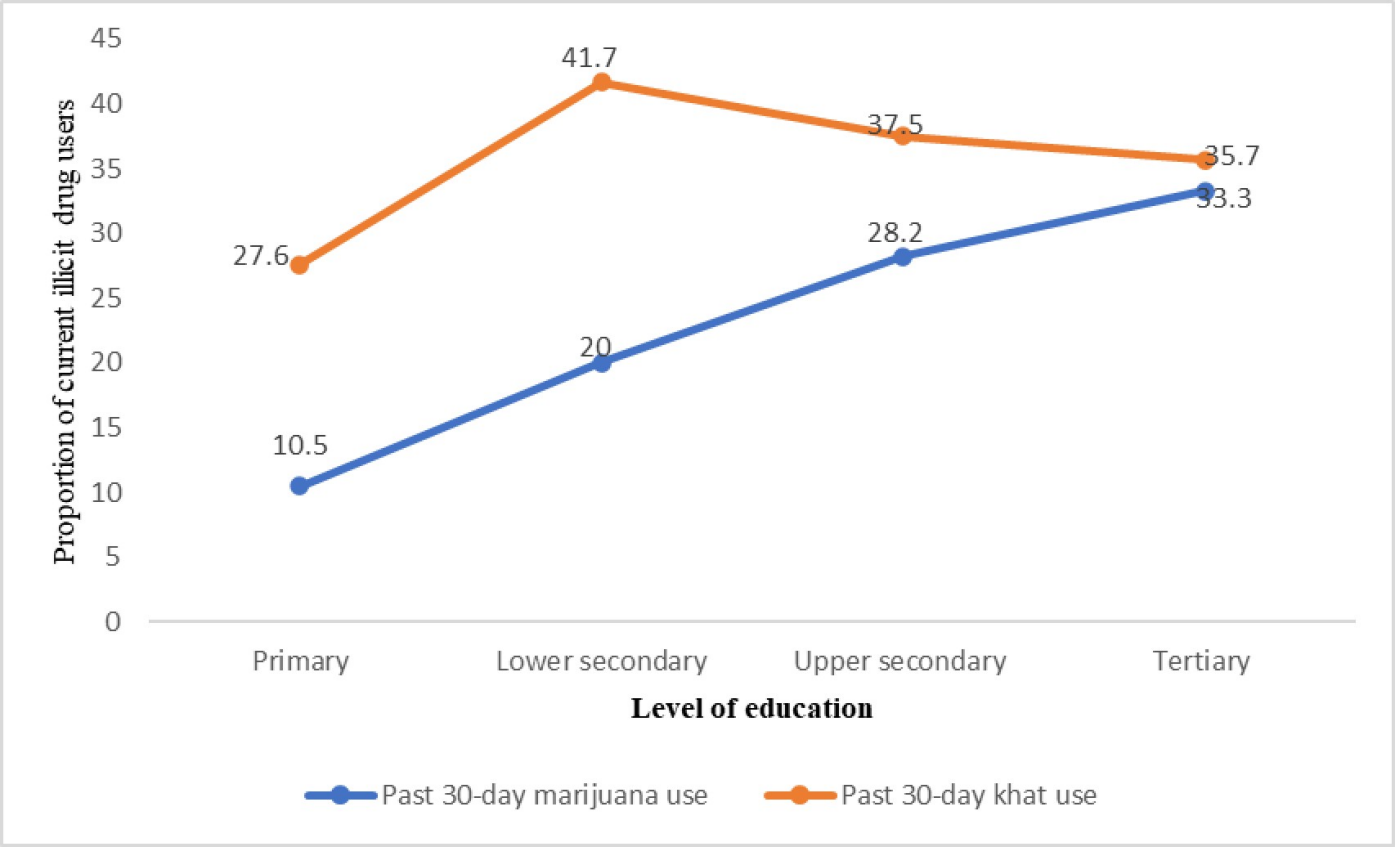
Past 30-day marijuana and khat use across level of education.

Past-30 days use of either drug was higher among past-year khat users (46%, n = 133) than marijuana users (36.9%, n = 97). Marijuana or khat use in the past 30 days was higher among out-of-school than in-school ABYM. We also observed a similar pattern with age and education: past 30-days use of marijuana or khat increased with increasing age (**Fig 3**) and level of education (**Fig 4**).

#### Parental attention among adolescent boys and young men

Table 4 shows the proportion of ABYM who reported that they always received general parental attention in the 30 days preceding the survey, across different background characteristics. Overall, parental attention was low across all parental attention attributes, ranging from 13% for “*being praised*” to 31.9% for “*being given advice and guidance*”. In general, parental attention was higher among ABYM who were in school than those who were out-of-school and decreased with increasing age and education levels, and this was true for all attributes.

**Table 4:** Percentage of adolescent boys and young men who reported that they always received parental attention in the 30 days preceding the survey, by selected parental attention attributes.

Background characteristics	Total	Being comforted	Being understood	Being listened to and given attention	Having open communication	Being shown affection	Spending time together with parents	Being praised	Being given advice and guidance	Being given money
**Overall**	2500	525 (21.0)	585 (23.4)	605 (24.2)	603 (24.1)	618 (24.7)	451 (18.0)	331 (13.2)	798 (31.9)	236 (9.4)
**Schooling status**										
In-school	1869	426 (22.8)	490 (26.2)	508 (27.2)	505 (27.0)	516 (27.6)	394 (21.1)	289 (15.5)	665 (35.6)	219 (11.7)
Out-of-school	631	99 (15.7)	95 (15.1)	97 (15.4)	98 (15.5)	102 (16.2)	57 (9.0)	42 (6.7)	133 (21.1)	17 (2.7)
**Age-group**										
10–14	483	143 (29.6)	171 (35.4)	187 (38.7)	171 (35.4)	194 (40.2)	138 (28.6)	120 (24.8)	208 (43.1)	82 (17.0)
15–19	1182	241 (20.4)	256 (21.7)	260 (22.0)	266 (22.5)	256 (21.7)	215 (18.2)	136 (11.5)	374 (31.6)	115 (9.7)
20–24	835	141 (16.9)	158 (18.9)	158 (18.9)	166 (19.9)	168 (20.1)	98 (11.7)	75 (8.9)	216 (25.9)	39 (4.7)
**Education level**										
Primary	421	121 (28.7)	138 (32.8)	147 (34.9)	133 (31.6)	154 (36.6)	111 (26.4)	88 (20.9)	164 (39.0)	65 (15.4)
Lower secondary	826	191 (23.1)	217 (26.3)	226 (27.4)	224 (27.1)	228 (27.6)	186 (22.5)	131 (15.9)	317 (38.4)	101 (12.2)
Upper secondary	397	75 (18.9)	87 (21.9)	87 (21.9)	97 (24.4)	83 (20.9)	66 (16.6)	43 (10.8)	121 (30.5)	39 (9.8)
Tertiary	225	39 (17.3)	48 (21.3)	48 (21.3)	51 (22.7)	51 (22.7)	31 (13.8)	27 (12.0)	63 (28.0)	14 (6.2)

#### Parental attention and illicit drug use among adolescent boys and young men

Table 5 shows the proportion of current illicit drug users who reported always being given attention in the 30 days preceding the survey, stratified by whether or not they were current tobacco, marijuana or khat users. In the majority of cases, current illicit drug users reported that they received any form of parental attention for some of the time but only ~10% reported that they always received parental attention across the board. For instance, only 11.8% (n = 2) of current tobacco users; 13.4% (n = 13) of current marijuana users, and 10.5% (n = 14) of current khat users reported that they were always given advice and guidance (on any subject) by their parents. On average, 71% of current illicit drug users reported that they had never been given money by their parents; 68% had never been praised by their parents; 55% had never spent time together with their parents, while 52% had never been comforted by their parents.

**Table 5:** Parental attention in the past 30 days among adolescent boys and young men who are current tobacco, marijuana and khat users.

Parental attention attributes (past 30 days)	Current users (past 30 days)		
	Tobacco use N = 17	Use of marijuana N = 97	Use of khat N = 133
**During the past 30 days, how often did your parents or guardians comfort you?**			
Always	1 (5.9)	9 (9.3)	10 (7.5)
Sometimes	7 (41.2)	36 (37.1)	56 (42.1)
Never	9 (52.9)	52 (53.6)	67 (50.4)
**During the past 30 days, how often did your parents or guardians understand you?**			
Always	2 (11.8)	10 (10.3)	10 (7.5)
Sometimes	11 (64.7)	49 (50.5)	71 (53.4)
Never	4 (23.5)	38 (39.2)	52 (39.1)
**During the past 30 days, how often did your parents or guardians listen to you?**			
Always	2 (11.8)	10 (10.3)	12 (9.0)
Sometimes	12 (70.6)	44 (45.4)	63 (47.4)
Never	3 (17.6)	43 (44.3)	58 (43.6)
**During the past 30 days, how often did your parents or guardians have open communication with you?**			
Always	2 (11.8)	6 (6.2)	7 (5.3)
Sometimes	11 (64.7)	51 (52.6)	75 (56.4)
Never	4 (23.5)	40 (41.2)	51 (38.3)
**During the past 30 days, how often did your parents or guardians show you affection?**			
Always	3 (17.7)	9 (9.3)	17 (12.8)
Sometimes	6 (35.3)	39 (40.2)	60 (45.1)
Never	8 (47.1)	49 (50.5)	56 (42.1)
**During the past 30 days, how often did your parents or guardians spend time together with you?**			
Always	1 (5.9)	6 (6.2)	8 (6.0)
Sometimes	10 (58.8)	28 (28.9)	38 (28.6)
Never	6 (35.3)	63 (65.0)	87 (65.4)
**During the past 30 days, how often did your parents or guardians praise you?**			
Always	0 (0.0)	4 (4.1)	5 (3.8)
Sometimes	5 (29.4)	24 (24.7)	43 (32.3)
Never	12 (70.6)	69 (71.1)	85 (63.9)
**During the past 30 days, how often did your parents or guardians advise and guide you?**			
Always	2 (11.8)	13 (13.4)	14 (10.5)
Sometimes	12 (70.6)	56 (57.7)	80 (60.2)
Never	3 (17.6)	28 (28.9)	39 (29.3)
**During the past 30 days, how often did your parents or guardians give you money?**			
Always	0 (0.0)	4 (4.1)	2 (1.5)
Sometimes	4 (23.5)	27 (27.8)	38 (28.6)
Never	13 (76.5)	66 (68.0)	93 (69.9)

## Discussion

Our study on the use of tobacco and illicit drugs among adolescent boys and young men in Kampala, Uganda, showed that: a) use of illicit drugs was higher among out-of-school than in-school ABYM and increased with increasing age and education levels; b) parental attention was lower among out-of-school than in-school ABYM and decreased with increasing age and education levels; and c) parental attention was low among current illicit drug users. Collectively, these findings suggest that use of illicit drugs is common among ABYM but parental attention towards them is low. These findings suggest a need to focus parental attention efforts on the most common illicit drug users.

Our finding that use of illicit drugs is higher among out-of-school ABYM is consistent with findings from Uganda, South Africa and the United States, which found that being away from school was a unique predictor of adolescent illicit drug use [25,38–40]. Most likely, illicit drug use among out-of-school ABYM may be influenced by peer pressure, since they typically spend more time around their peers than parents [41,42]. Perhaps, out-of-school ABYM continue to use illicit drugs to cope with any recurring unmet emotional, psychological or economic needs [25,43]. This is plausible, given the fact that in our study, parental attention was lower among out-of-school ABYM. Since ABYM who are out-of-school may not necessarily always be around their parents, they are more likely to have greater degree of autonomy and freedom to use illicit drugs. Our findings thus point to the likely worrying health trends among out-of-school ABYM in general, and call for a need to give them increasing attention.

We found that both past-year and current illicit drug use among ABYM increased with increasing age. Our findings are consistent with findings from the United States which found an increase in use of any tobacco products and marijuana with age [38,44], and resonate with findings from England which reported a similar pattern [45]. Altogether, these studies confirm that age is a key influencer for illicit drug use, especially among older adolescents and young men. When we further stratified our analysis by age-group, we found that use of illicit drugs sharply increases among ABYM transitioning from 15–24 years. The transition to teenage years is often a time for exploration, filled with the desire to try something new or risky. The adolescents who perceive little risk in using drugs will more readily use them [46]. While it is probable that the transition from adolescence to young adulthood is associated with increased frequency of drug use [47], we are not certain of what could have been solely responsible for the increase in use of illicit drugs among ABYM aged 15 years and older in our study, and therefore more research is warranted on this phenomenon. However, our study advances the understanding of illicit drug use among adolescents, by presenting data on the very young adolescents aged 10–14 years. Our findings thus underscore the critical importance of prevention strategies that target the young adolescent boys, which are aimed at empowering them to desist from illicit drug use initiation which would otherwise lead into future dependence.

Our study also found that illicit drug use among ABYM increased with increasing level of education. These findings are consistent with findings from Kenya, Brazil and the United States which found that illicit drug use increased as adolescents progressed through school [48–50]. Some literature suggests that increase in illicit drug use is because students are trying to cope with the intense academic pressure and expectations onto them for good school performance [51,52]. These findings suggest that schools are perhaps not drug-free environments, and call for a need to focus on instilling life skills among ABYM, for them to be able to keep up with academic demands without having to use illicit drugs. However, our study did not assess any association between illicit drug use and academic performance, and therefore, we cannot attribute the increased use of illicit drugs among better-educated ABYM to academic pressure in schools. Nevertheless, our findings suggest a need for further research to fully understand why better-educated ABYM were more likely to use illicit drugs than those with no or lower education.

### Study limitations and strengths

Our study has some limitations and strengths. In the first place, we did not aim to determine if there was a dose-response effect between parental attention and use or non-use of illicit drugs, and thus, our paper does not present any statistical tests for associations, which we believe could have improved the strength of our findings. Therefore, we do not know if it was due to lack of parental attention that ABYM engaged in the use of illicit drugs or whether or not it was the use of illicit drugs that led parents to withdraw their attention from the users of illicit drugs. The second limitation is that our sample was composed of out-of-school ABYM who were interviewed from their workplaces and therefore, it is likely that this group may not be representative of all the out-of-school ABYM in Kampala. We do not know if reporting use of illicit drugs would have been mediated by the place of interview–for instance, if the use of illicit drugs among out-of-school ABYM would have been different if the interviews were conducted at household level. Similarly, given that in-school ABYM were also interviewed from home, it is likely that they could have under-reported their use of illicit drugs, and thus our findings may be an underestimation of the true prevalence. There could also have been a potential recall bias from the participants on their tobacco and illicit drug use within the past 12 months, and therefore, our findings may still not be a sufficient representation of prevalence of use of these substances within this period. Lastly, given the fact that our study was not powered to detect if any association exists between the level of parental attention and illicit drug use, further research to determine if such an association exists is warranted.

Despite the above-mentioned limitations, our findings point to an important relationship: parental attention was higher among those who were in-school and those that were younger (e.g. 10–14 years) than those who were out-of-school or older. These groups were less likely to use illicit drugs. Thus, the limited parental attention paid to groups of ABYM that were more likely to use illicit drugs calls for intensified efforts for parents directed to out-of-school ABYM, older adolescent boys and young men, and better-educated ABYM. Besides, our study enrolled those aged 10–14 years who are usually missed in most conventional surveys. Therefore, we believe that our findings can help to inform programming approaches that can help to target adolescent boys before they reach the age at which illicit drug use shoots up and therefore prevent the increasing use of illicit drugs among ABYM not only in Kampala but in other areas of the world.

## Conclusion

Our study shows that use of illicit drugs is a growing concern among ABYM but parental attention among current illicit drug users is low. In general, use of illicit drugs increased with increasing age and highest level of education attained but parental attention decreased with increasing age and education level. Parental attention was also higher among in-school than out-of-school ABYM yet use of illicit drugs was higher among those who were out-of-school. While it is not easy to tell whether or not the lack of parental attention precipitated the use of tobacco or other illicit drugs among ABYM, these findings point to a need to design interventions intended to empower parents to be more involved in the growth and development of ABYM. Interventions that target out-of-school and older ABYM with appropriate harm reduction messages are also urgently needed to reduce the use of tobacco or other illicit drugs in this population.

## Supporting information

S1 File Use of tobacco and other illicit drugs study dataset. (DTA)

S2 File Survey questionnaire. (DOC)

## Abbreviations

ABYM: Adolescent Boys and Young Men
CDC: Centers for Disease Control and Prevention
GSHS: Global School-based Student Health Survey
LQAS: Lot Quality Assurance Sampling
WHO: World Health Organization
