# Supplementary material for: Use of tobacco and other illicit drugs among adolescent boys and young men in Kampala, Uganda: A result of low parental attention?
Source: PLoS One. 2024 Mar 26;19(3):e0297163. doi: 10.1371/journal.pone.0297163 (PMC10965093; doi:10.1371/journal.pone.0297163)
Supplement: S2 File — (DOC) [file pone.0297163.s002.doc]

**Perception of Risk, Risk-taking Behaviors and Health Needs of Adolescent Boys and Young Men in Kampala, Uganda**

**QUANTITATIVE QUESTIONNARIE**

**November 2019**

| **Demographic Information** |  |  |  |
| --- | --- | --- | --- |
| **Question** | **Code** | **Question** | **Code** |
| What is your date of birth?  _____day  ______month  ______year | **C1**  **C2**  **C3** | How old are you?  ___________years | **C4** |
| Are you in school or out of school?  In school==1  Out of school==2  If Out of School, have you ever been to school?  Yes==1  No==2 | **C5**  **C5a** | Which class are you in?  Not in school==1  P4 – P5==2  P6 – P7==3  S1 – S2==4  S3 – S4==5  S5 – S6==6  Post-secondary education (university, tertiary)==7 | **C6** |
| What is your tribe?  Baganda==1  Banyankore==2  Iteso==3  Lugbara/Madi==4  Basoga==5  Langi==6  Bakiga==7  Karimojong==8  Acholi==9  Bagisu/Sabiny==10  Alur/Jopadhola==11  Banyoro==12  Batoro==13  Other==14  Refused==15  Other specify | **C7**  **C7a** | Which division of Kampala do you reside?  Central division==1  Makindye division==2  Nakawa division==3  Kawempe division==4  Rubaga division==5 | **C8** |
| **Section 1: Alcohol** | | | |
| **The next questions ask about the consumption of alcohol. A standard drink of alcohol is 285ml of beer, or a 120 ml glass of wine, or a tot of whisky, waragi or gin.** | | | |
| Have you **ever** consumed any alcohol such as beer, wine, spirits,waragi, malwa or any other local brews?  Yes==1  No==2 | **A1** | Have you consumed any alcohol such as beer, wine, spirits,waragi, malwa or any other local brews within the past 12 months?  Yes==1  No==2 | **A2** |
| During the past 12 months, **how frequently** have you had at least one standard alcoholic drink such as beer, wine, spirits, waragi, malwa or any other local brews?  Never==0  Daily==1  5-6 days per week==2  3-4 days per week==3  1-2 days per week==4  1-3 days per month==5  Less than once a month==6 | **A3** | Have you consumed any alcohol within the past 30 days?  Yes==1  No==2 | **A4** |
| During the past 30 days, on how many occasions did you have at least one standard drink such as beer, wine, spirits,waragi, malwa or any other local brews? [indicate 0 if none]  _________Number | **A5** | During the past 30 days, when you drank alcohol, how many standard drinks on average did you have during one drinking session? [indicate 0 if none]  _________Number | **A6** |
| During the past 30 days, when you drank alcohol, what was the largest number of standard drinks you had on a single occasion, counting all types of alcoholic drinks together? [indicate 0 if none]  _________Number | **A7** | During the past 30 days, how many times did you have 6 or more standard drinks during one drinking session? [indicate 0 if none]  _________Number | **A8** |
| During the past 30 days, how often did you see any alcohol advertisements?  Never==1  Rarely==2  Sometimes==3  Almost daily==4  Daily==5 | **A9** | How many of your friends frequently drink 5 or more drinks on one occasion?  None==1  A few==2  Some==3  Most==4  All==5 | **A10** |
| Staggering when walking, not being able to speak right, and throwing up are some signs of being really drunk.  How old were you the first time you drank so much alcohol that you were really drunk?  I have never drank so much alcohol that I was really drunk==1  7 years old or younger==2  8 or 9 years old==3  10 or 11 years old==4  12 or 13 years old==5  14 or 15 years old==6  16 or 17 years old==7  18 years old or older==8 | **A11** | Staggering when walking, not being able to speak right, and throwing up are some signs of being really drunk.  During the past 30 days, how many times did you drink so much alcohol that you were really drunk?  0 times==1  1 or 2 times==2  3 to 9 times==3  10 or more times==4 | **A12** |
| During the past 30 days, how many times did you get into trouble with your family or friends, miss school or get into fights as a result of drinking alcohol?  0 times==1  1 or 2 times==2  3 to 9 times==3  10 or more times==4 | **A13** | Which of your parents or guardians drink alcohol?  Neither==1  My father or male guardian==2  My mother or female guardian==3  Both==4  I do not know==5 | **A14** |
| Where were you the last time you had a drink of alcohol?  I have never had a drink of alcohol==1  At home==2  At someone else’s home==3  At school==4  Out on the street in a park or in some other open area==5  At a bar, pub, or disco==6  In a restaurant==7  Some other place==8 | **A15** | With whom do you usually drink alcohol?  I do not drink alcohol==1  With my friends==2  With my family==3  With persons I have just met==4  I usually drink alone==5 | **A16** |
| Are you allowed to drink at home?  I do not drink alcohol==1  Yes==2  No==3 | **A17** | During the past 30 days, did anyone refuse to sell to you alcohol because of your age  I did not try to buy alcohol during the past 30 days==1  Yes, someone refused to sell me alcohol because of my age==2  No, my age did not keep me from getting alcohol==3 | **A18** |
| Do any of your brothers and sisters drink alcohol?  I do not have brothers or sisters==1  Yes==2  No==3  I don’t know==4 | **A19** | How many of your friends drink alcohol?  None==1  A few==3  Some==4  Most==5  All==6 | **A20** |
| Do your parents or guardians know that you drink alcohol?  I do not drink alcohol==1  Yes==2  No==3  I do not know==4 | **A21** |  |  |
| **Role of the Media and Advertising** |  |  |  |
| When you watch television, videos, or movies, how often do you see actors drinking alcohol?  I do not watch television, videos, or movies==1  Never==2  Rarely==3  Sometimes==4  Most of the time==5  Always**==**6 | **A22** | When you go to sports events, fairs, concerts, community events, or social gatherings how often do you see advertisements for alcohol?  I do not go to sports events, fairs, concerts, community events, or social gatherings==1  Never==2  Rarely==3  Sometimes==4  Most of the time==5  Always**==**6 | **A23** |
| During the past 30 days, how many advertisements for alcohol have you seen when you watched television?  I have not watched television during the past 30 days==1  A lot==2  A few==3  None==4 | **A24** | During the past 30 days, how many advertisements for alcohol have you seen on billboards?  I have not seen a billboard during the past 30days==1  A lot==2  A few==3  None==4 | **A25** |
| During the past 30 days, how many advertisements for alcohol have you seen in newspapers or magazines?  I have not seen a newspaper or magazine during the past 30 days==1  A lot==2  A few==3  None==4 | **A26** | During the past 30 days, how many advertisements for alcohol have you seen on the internet?  I have not used the internet during the past 30 days==1  A lot==2  A few==3  None==4 | **A27** |
| Do you have something, such as a t-shirt, pen, backpack, or other item, with an alcohol brand logo on it?  Yes==1  No==2 | **A28** | How difficult do you think it would be for you to limit your alcohol consumption to 2 standard drinks or less per day?  I do not drink alcohol==1  Impossible==2  Very difficult==3  Fairly difficult==4  Fairly easy==5  Very easy==6  I do not know==7 | **A29** |
| On a scale of 0-100, can you rate how certain you are that you can limit your alcohol consumption to 2 standard drinks or less per day where  0-----Cannot at all  50-----Can do moderately  100-----Highly certain can do  Rate___________ | **A30** | Do you think it would be beneficial to your health for you to limit your alcohol consumption to less than 2 standard drinks per day?  Yes==1  No==2  If yes, how beneficial to your health would it be for you to limit your alcohol consumption to less than 2 standard drinks per day?  Very beneficial==1  Moderately beneficial==2  A little beneficial==3 | **A31** |
| **Knowledge, Attitudes, Skills, and Sources of Information** | | |  |
| If one of your best friends offered you a drink of alcohol, would you drink it?  Definitely not==1  Probably not==2  Probably yes==3  Definitely yes==4 | **A32** | How difficult do you think it would be for you to get alcohol, such as beer, wine, waragi if you wanted to?  Impossible==1  Very difficult==2  Fairly difficult==3  Fairly easy==4  Very easy==5  I do not know==6 | **A33** |
| During this school year, were you taught in any of your classes the problems associated with drinking alcohol?  Yes==1  No==2  I do not know==3  Not in school==4 | **A34** | During this school year, were you taught in any of your classes the effects of alcohol use on decision making?  Yes==1  No==2  I do not know==3  Not in school==4 | **A35** |
| During this school year, were you taught in any of your classes how to tell someone you did not want to drink alcohol?  Yes==1  No==2  I do not know==3  Not in school==4 | **A36** |  |  |

**Section 2: Dietary Behaviors**

| **Question** | **Code** | **Question** | **Code** | |
| --- | --- | --- | --- | --- |
| During the past 30 days, how often did you eat breakfast?  Never==1  Rarely==2  Sometimes==3  Most of the time==4  Always==5 | **F1** | How do you describe your weight?  Very underweight==1  Slightly underweight==2  About the right weight==3  Slightly overweight==4  Very overweight==5 | **F2** | |
| Which of the following are you trying to do about your weight?  I am not trying to do anything about my weight==1  Lose weight==2  Gain weight==3  Stay the same weight==4 | **F3** | During the past 12 months, have you been weighed and measured?  Yes==1  No==2  If yes, what is your weight? ________kg  What is your height? __________(meters) | **F4**  **F4a**  **F4b** | |
| What is the main reason you do not eat breakfast?  I always eat breakfast==1  I do not have time for breakfast==2  I cannot eat early in the morning==3  There is not always food in my home==4  Some other reason==5 | **F5** | During the past 30 days, did you exercise to lose weight or to keep from gaining weight?  Yes==1  No==2 | **F6** | |
| During the past 30 days, did you take any diet pills, powders or liquids without a doctor’s advice to lose weight or to keep from gaining weight  Yes==1  No==2 | **F7** | During the past 30 days, did you eat less food, fewer calories or foods low in fat to lose weight or to keep from gaining weight?  Yes==1  No==2 | **F8** | |
| During the past 30 days, did you go without eating for 24 hours or more (also called fasting) to lose weight or to keep from gaining weight?  Yes==1  No==2 | **F9** | During the past 30 days, did you vomit or take laxatives to lose weight or to keep from gaining weight?  Yes==1  No==2 | **F10** | |
| During the past 30 days, did you exercise to gain weight?  Yes==1  No==2 | **F11** | During the past 30 days did you eat more food, more calories or foods high in fat to gain weight?  Yes==1  No==2 | **F12** | |
| During the past 30 days, did you take any diet pills, powders or liquids without a doctor’s advice to lose weight or to gain weight  Yes==1  No==2 | **F13** | During the past 30 days, how often did you bring your lunch to school?  Never==1  Rarely==2  Sometimes==3  Most of the time==4  Always==5 | **F14** | |
| During the past 30 days, how often was breakfast offered at school?  Never==1  Rarely==2  Sometimes==3  Most of the time==4  Always==5  Not in school==6 | **F15** | During the past 30 days, how often was lunch offered at school?  Never==1  Rarely==2  Sometimes==3  Most of the time==4  Always==5  Not in school==6 | **F16** | |
| During the past 30 days, how many times per day did you usually drink milk or eat milk products such as ice cream or yoghurt?  I did not drink milk or eat milk products during the past 30 days==1  Less than one time per day==2  1 time per day==3  2 times per day==4  3 times per day==5  4 times per day==6  5 or more times per day==7 | **F17** | During the past 30 days, how many times per day did you usually eat salty foods such as chips, crisps, sausages?  I did not salty foods==1  Less than one time per day==2  1 time per day==3  2 times per day==4  3 times per day==5  4 times per day==6  5 or more times per day==7 | **F18** | |
| During the past 30 days, how many times per day did you usually eat foods high in fat such as ghee, butter?  I did not foods high in fat==1  Less than one time per day==2  1 time per day==3  2 times per day==4  3 times per day==5  4 times per day==6  5 or more times per day==7 | **F19** | During the past 30 days, how many times per day did you usually drink fruit juice such as butunda, munanansi?  I did not drink fruit juice in the past 30 days==1  Less than one time per day==2  1 time per day==3  2 times per day==4  3 times per day==5  4 times per day==6  5 or more times per day==7 | **F20** | |
| In a typical week, on how many days do you eat fruit?  _______days | **F21** | How many servings of fruit do you eat on one of those days? (show card)  _______servings | **F22** | |
| In a typical week, on how many days do you eat vegetables?  _______days | **F23** | How many servings do you eat on one of those days? (show card)  _______servings | **F24** | |
| How difficult do you think it would be for you to eat at least 5 servings of fruit and/ or vegetables per day?  I do not eat fruits and vegetables==1  Impossible==2  Very difficult==3  Fairly difficult==4  Fairly easy==5  Very easy==6  I do not know==7 | **F25** | On a scale of 0-100, can you rate how certain you are that you can eat at least 5 servings of fruits and/ or vegetables per day where  0-----Cannot at all  50-----Can do moderately  100-----Highly certain can do  Rate___________ | | **F26** |
| Do you think it would be beneficial to your health for you to eat at least 5 servings of fruits and/ or vegetables per day?  Yes==1  No==2  If yes, how beneficial to your health would it be for you to eat at least 5 servings of fruits and/ or vegetables per day?  Very beneficial==1  Moderately beneficial==2  A little beneficial==3 | **F27a**  **F27b** |  | |  |
| **Role of the Media and Advertising** | | | | |
| **The next 7 questions ask about how carbonated soft drinks such as Coca Cola, Fanta, Pepsi, Riham Cola and foods from fast food restaurants such as Javas, KFC, Chicken tonight are advertised and sold** | | | | |
| When you watch television, videos or movies, how often do you see advertisements for carbonated soft drinks like CocaCola and Fanta or fast foods like KFC?  I do not watch TV, Videos or movies  Never==1  Rarely==2  Sometimes==3  Most of the time==4  Always==5 | **F28** | During the past 30 days, how many advertisements for carbonated soft drinks like CocaCola and Fanta or fast foods like KFC did you see when you watched TV?  I did not watch TV during the past 30 days==1  A lot==2  A few==3  None==4 | **F29** | |
| During the past 30 days, how many advertisements for carbonated soft drinks or fast foods did you see on the internet?  I did not use the internet during the past 30 days==1  A lot==2  A few==3  None==4 | **F30** | During the past 30 days, how many text messages or mobile phone calls did you get that encouraged you to go to a carbonated soft drink or fast food company website?  I did get any messages or calls during the past 30 days==1  A lot==2  A few==3  None==4 | **F31** | |
| Can you buy carbonated drinks or get them form free in your school?  Yes==1  No==2  Not in school==3 | **F32** | Can you buy fast foods or get them for free in your school?  Yes==1  No==2  Not in school==3 | **F32** | |
| During the past 30 days, how many advertisements for carbonated soft drinks or fast foods did you see in your school?  I did not see any advertisements for carbonated soft drinks or fast foods in my school==1  A lot==2  A few==3  None==4  Not in school==5 | **F33** | How often do you buy and eat street food like chips, sausages, rolex etc?  Never==1  Rarely==2  Sometimes==3  Most of the time==4  Always==5 | **34** | |
| **Knowledge, Attitudes, Skills and Sources of Information** | | | | |
| During this school year, were you taught in any of your classes the benefits of eating healthy?  Yes==1  No==2  I do not know==3  Not in school==4 | **F35** | During this school year, were you taught in any of your classes the benefits of eating more fruits and vegetables?  Yes==1  No==2  I do not know==3  Not in school==4 | **F36** | |
| During this school year, were you taught in any of your classes how to safely prepare or store food?  Yes==1  No==2  I do not know==3  Not in school==4 | **F37** | During this school year, were you taught in any of your classes healthy ways to gain weight?  Yes==1  No==2  I do not know==3  Not in school==4 | **F38** | |
| During this school year, were you taught in any of your classes healthy ways to lose weight?  Yes==1  No==2  I do not know==3  Not in school==4 | **F39** |  |  | |

**Section 3: Illicit drug use**

| **Question** | **Code** | **Question** | **Code** |
| --- | --- | --- | --- |
| During your life, how many times have you got into trouble with your family or friends, missed school or got into fights as a result of using drugs?  0 times==1  1 or 2 times==2  3 to 9 times==3  10 to 19 times==4  20 or more times==5 | **D1** | During your life, how many times have you used marijuana (enjaga)?  0 times==1  1 or 2 times==2  3 to 9 times==3  10 to 19 times==4  20 or more times==5  If ≥1, how old were you when you first used marijuana?  _________ years  If ≥1, how long ago did you first use marijuana?  _________ years  If ≥1, who prompted/ initiated you into using marijuana the first time?  No one, I did it myself==1  A close friend==2  A relative==3  Someone at work==4  Other==5  Specify other_____________________  How much money did you pay for a session of marijuana?  _______________shs | **D2a**  **D2b**  **D2c**  **D2d**  **D2e**  **D2f** |
| During the past 12 months, how many times have you used marijuana (enjaga)?  0 times==1  1 or 2 times==2  3 to 9 times==3  10 to 19 times==4  20 or more times==5 | **D3** | During the past 30 days, how many times have you used marijuana?  0 times==1  1 or 2 times==2  3 to 9 times==3  10 to 19 times==4  20 or more times==5 | **D4** |
| How difficult do you think it would be for you to get marijuana?  Impossible==1  Very difficult==2  Fairly difficult==3  Fairly easy==4  Very easy==5  I do not know==6 | **D6** | During your life, how many times have you used khat (mairungi)?  0 times==1  1 or 2 times==2  3 to 9 times==3  10 to 19 times==4  20 or more times==5  If ≥1, how old were you when you first used khat (mairungi)?  _________ years  If ≥1, how long ago did you first use khat (mairungi)?  _________ years  If ≥1, who prompted/ initiated you into using khat (mairungi) the first time?  No one, I did it myself==1  A close friend==2  A relative==3  Someone at work==4  Other==5  Specify other_____________________  How much money did you pay for a session of khat (mairungi)?  _______________shs | **D7a**  **D7b**  **D7c**  **D7d**  **D7e**  **D7f** |
| During the past 12 months, how many times have you used khat (mairungi)?  0 times==1  1 or 2 times==2  3 to 9 times==3  10 to 19 times==4  20 or more times==5 | **D8** | During the past 30 days, how many times have you used khat (mairungi)?  0 times==1  1 or 2 times==2  3 to 9 times==3  10 to 19 times==4  20 or more times==5 | **D9** |
| How difficult do you think it would be for you to get khat (mairungi)?  Impossible==1  Very difficult==2  Fairly difficult==3  Fairly easy==4  Very easy==5  I do not know==6 | **D10** | During your life, how many times have you used amphetamines or methamphetamines?  0 times==1  1 or 2 times==2  3 to 9 times==3  10 to 19 times==4  20 or more times==5  If ≥1, how old were you when you first used amphetamines or methamphetamines?  _________ years  If ≥1, how long ago did you first use amphetamines or methamphetamines?  _________ years  If ≥1, who prompted/ initiated you into using amphetamines or methamphetamines the first time?  No one, I did it myself==1  A close friend==2  A relative==3  Someone at work==4  Other==5  Specify other_____________________  How much money do you pay for a session of amphetamines or methamphetamines?  _______________shs | **D10a**  **D10b**  **D10c**  **D10d**  **D10e**  **D10f** |
| During the past 12 months, how many times have you used amphetamines or methamphetamines?  0 times==1  1 or 2 times==2  3 to 9 times==3  10 to 19 times==4  20 or more times==5 | **D11** | During the past 30 days, how many times have you used amphetamines or methamphetamines?  0 times==1  1 or 2 times==2  3 to 9 times==3  10 to 19 times==4  20 or more times==5 | **D12** |
| How difficult do you think it would be for you to get amphetamines or methamphetamines?  Impossible==1  Very difficult==2  Fairly difficult==3  Fairly easy==4  Very easy==5  I do not know==6 | **D13** | During your life, how many times have you used cocaine?  0 times==1  1 or 2 times==2  3 to 9 times==3  10 to 19 times==4  20 or more times==5  If ≥1, how old were you when you first used cocaine?  _________ years  If ≥1, how long ago did you first use cocaine?  _________ years  If ≥1, who prompted/ initiated you into using cocaine the first time?  No one, I did it myself==1  A close friend==2  A relative==3  Someone at work==4  Other==5  Specify other_____________________  How much money did you pay for a session of cocaine?  _______________shs | **D14a**  **D14b**  **D14c**  **D14d**  **D14e**  **D14f** |
| During the past 12 months, how many times have you used cocaine?  0 times==1  1 or 2 times==2  3 to 9 times==3  10 to 19 times==4  20 or more times==5 | **D15** | During the past 30 days, how many times have you used cocaine?  0 times==1  1 or 2 times==2  3 to 9 times==3  10 to 19 times==4  20 or more times==5 | **D16** |
| How difficult do you think it would be for you to get cocaine?  Impossible==1  Very difficult==2  Fairly difficult==3  Fairly easy==4  Very easy==5  I do not know==6 | **D17** | During your life, how many times have you used solvents or inhalants such as petrol or jet fuel?  0 times==1  1 or 2 times==2  3 to 9 times==3  10 to 19 times==4  20 or more times==5  If ≥1, how old were you when you first used solvents or inhalants such as petrol or jet fuel?  _________ years  If ≥1, how long ago did you first use solvents or inhalants such as petrol or jet fuel?  _________ years  If ≥1, who prompted/ initiated you into using solvents or inhalants such as petrol or jet fuel the first time?  No one, I did it myself==1  A close friend==2  A relative==3  Someone at work==4  Other==5  Specify other_____________________  How much money did you pay for a session of solvents or inhalants such as petrol or jet fuel?  _______________shs | **D18a**  **D18b**  **D18c**  **D18d**  **D18e**  **D18f** |
| During the past 12 months, how many times have you used solvents or inhalants such as petrol or jet fuel?  0 times==1  1 or 2 times==2  3 to 9 times==3  10 to 19 times==4  20 or more times==5 | **D19** | During the past 30 days, how many times have you used solvents or inhalants such as petrol or jet fuel?  0 times==1  1 or 2 times==2  3 to 9 times==3  10 to 19 times==4  20 or more times==5 | **D20** |
| How difficult do you think it would be for you to get solvents or inhalants such as petrol or jet fuel?  Impossible==1  Very difficult==2  Fairly difficult==3  Fairly easy==4  Very easy==5  I do not know==6 | **D21** | During your life, how many times have you used ecstasy?  0 times==1  1 or 2 times==2  3 to 9 times==3  10 to 19 times==4  20 or more times==5  If ≥1, how old were you when you first used ecstasy?  _________ years  If ≥1, how long ago did you first use ecstasy?  _________ years  If ≥1, who prompted/ initiated you into using ecstasy the first time?  No one, I did it myself==1  A close friend==2  A relative==3  Someone at work==4  Other==5  Specify other_____________________  How much money did you pay for a session of ecstacy?  _______________shs | **D22a**  **D22b**  **D22c**  **D22d**  **D22e**  **D22f** |
| During the past 12 months, how many times have you used ecstasy?  0 times==1  1 or 2 times==2  3 to 9 times==3  10 to 19 times==4  20 or more times==5 | **D23** | During the past 30 days, how many times have you used ecstasy?  0 times==1  1 or 2 times==2  3 to 9 times==3  10 to 19 times==4  20 or more times==5 | **D24** |
| How difficult do you think it would be for you to get ecstasy?  Impossible==1  Very difficult==2  Fairly difficult==3  Fairly easy==4  Very easy==5  I do not know==6 | **D25** | During your life, how many times have you used heroine?  0 times==1  1 or 2 times==2  3 to 9 times==3  10 to 19 times==4  20 or more times==5  If ≥1, how old were you when you first used heroine?  _________ years  If ≥1, how long ago did you first use heroine?  _________ years  If ≥1, who prompted/ initiated you into using heroine the first time?  No one, I did it myself==1  A close friend==2  A relative==3  Someone at work==4  Other==5  Specify other_____________________  How much money did you pay for a session of heroine?  _______________shs | **D26a**  **D26b**  **D26c**  **D26d**  **D26e**  **D26f** |
| During the past 12 months, how many times have you used heroine?  0 times==1  1 or 2 times==2  3 to 9 times==3  10 to 19 times==4  20 or more times==5 | **D27** | During the past 30 days, how many times have you used heroine?  0 times==1  1 or 2 times==2  3 to 9 times==3  10 to 19 times==4  20 or more times==5 | **D28** |
| How difficult do you think it would be for you to get heroine?  Impossible==1  Very difficult==2  Fairly difficult==3  Fairly easy==4  Very easy==5  I do not know==6 | **D29** | During your life, how many times have you used a prescription drug without a doctor’s prescription?  0 times==1  1 or 2 times==2  3 to 9 times==3  10 to 19 times==4  20 or more times==5  If ≥1, how old were you when you first used a prescription drug without a doctor’s prescription?  _________ years  If ≥1, how long ago did you first use a prescription drug without a doctor’s prescription?  _________ years  If ≥1, who prompted/ initiated you into using a prescription drug without a doctor’s prescription the first time?  No one, I did it myself==1  A close friend==2  A relative==3  Someone at work==4  Other==5  Specify other_____________________  How much money did you pay for a prescription drug without a doctor’s prescription?  _______________shs | **D30a**  **D30b**  **D30c**  **D30d**  **D30e**  **D30f** |
| During the past 12 months, how many times have you used a prescription drug without a doctor’s prescription?  0 times  1 or 2 times  3 to 9 times  10 to 19 times  20 or more times | **D31** | During the past 30 days, how many times have you used a prescription drug without a doctor’s prescription?  0 times==1  1 or 2 times==2  3 to 9 times==3  10 to 19 times==4  20 or more times==5 | **D32** |
| How difficult do you think it would be for you to get a prescription drug without a doctor’s prescription?  Impossible==1  Very difficult==2  Fairly difficult==3  Fairly easy==4  Very easy==5  I do not know==6 | **D33** | During your life, how many times have you used a needle to inject any illegal drug into your body?  0 times==1  1 or 2 times==2  3 to 9 times==3  10 to 19 times==4  20 or more times==5  If≥1 have you ever shared a needle that had been used by another person to inject any illegal drug in your body?  Yes==1  No==2 | **D34a**  **D34b** |
| During this school year, were you taught in any of your classes the problems associated with using drugs such as marijuana, tobacco, cocaine?  Yes==1  No==2  I do not know==3  Not in school==4 | **D35** | Where can you obtain health services related to drug use if you required them?  Nearby clinic==1  Government health center==2  Private health center==3  Butabika==4  Other==5  Specify if other_______________ | **D36a**  **D36b** |

**Section 4: Tobacco**

| **Question** | **Code** | **Question** | **Code** |
| --- | --- | --- | --- |
| Have you ever tried or experimented with cigarettes smoking even one or two puffs?  Yes==1  No==2 | **T1** | How old were you when you first tried a cigarette?  I have never tried smoking a cigarette==1  7 years old or younger==2  8 or 9 years old==3  10 or 11 years old==4  12 or 13 years old==5  14 or 15 years old==6  16 years or older==7 | **T2** |
| During the past 30 days, on how many days did you smoke cigarettes?  0 days==1  1 or 2 days==2  3 to 5 days==3  6 to 9 days==4  10 to 19 days==5  20 to 29 days==6  All 30 days==7 | **T3** | Please think about the days that you smoked cigarettes during the past 30 days. How many cigarettes did you usually smoke per day?  I did not smoke cigarettes during the past 30 days==1  Less than 1 cigarette per day==2  1 cigarette per day==3  2 to 5 cigarettes per day==4  6 to 10 cigarettes per day==5  11 to 20 cigarettes per day==6  More than 20 cigarettes per day==7 | **T4** |
| Have you ever tried or experimented with any form of smoked tobacco products other than cigarettes such as shisha, cigars, or pipes?  Yes==1  No==2 | **T5** | During the past 30 days, did you use any form of smoked tobacco products other than cigarettes such as shisha, cigars, or pipes?  Yes==1  No==2 | **T6** |
| Do you ever smoke tobacco or feel like smoking tobacco first thing in the morning?  I don’t smoke tobacco==1  No I don’t smoke tobacco or feel like smoking tobacco first thing in the morning==2  Yes, I sometimes smoke tobacco or feel like smoking tobacco first thing in the morning==3  Yes, I always smoke tobacco or feel like smoking tobacco first thing in the morning==4 | **T7** | How soon after you smoke tobacco do you start to feel a strong desire to smoke again that is hard to ignore?  I don’t smoke tobacco==1  I never feel a strong desire to smoke again after smoking tobacco==2  Within 60 minutes==3  1 to 2 hours==4  More than 2 hours to 4 hours==5  More than 4 hours but less than one full day==6  1 to 3 days==7  4 days or more==8 | **T9** |
| Have you ever tried or experimented with smokeless tobacco products such as bugolo, etabe, kuba or gutka?  Yes==1  No==2 | **T10** | How old were you when you first tried a smokeless tobacco product such as bugolo, etabe, kuba or gutka?  I have never tried any smokeless tobacco product==1  7 years old or younger==2  8 or 9 years old==3  10 or 11 years old==4  12 or 13 years old==5  14 or 15 years old==6  16 years or older==7 | **T11** |
| During the past 30 days, did you use any form of smokeless tobacco product such as bugolo, etabe, kuba or gutka?  Yes==1  No==2 | **T12** | The next questions ask about your feelings towards stopping smoking |  |
| Do you want to stop smoking now?  I have never smoked==1  I don’t smoke now==2  Yes==3  No==4 | **T13** | During the past 12 months, did you ever try to stop smoking?  I have never smoked  I did not smoke during the past 12 months  Yes==1  No==2 | **T14** |
| Do you think you would be able to stop smoking if you wanted to?  I have never smoked==1  I don’t smoke now==2  Yes==3  No==4 | **T15** | Have you ever received help or advice to help you stop smoking?  I have never smoked==1  Yes, from a program or a professional==2  Yes, from a friend==3  Yes, from a family member==4  Yes, from both programs or professionals and from friends or family members==5  No==6 | **T16** |
| During the past 7 days, on how many days has anyone smoked inside your home in your presence?  0 days==1  1 to 2 days==2  3 to 4 days==3  5 to 6 days==4  7 days==5 | **T17** | During the past 7 days, on how many days has anyone smoked in your presence, inside any enclosed place other than your home such as a school, shop, restaurant, shopping mall, movie theater?  0 days==1  1 to 2 days==2  3 to 4 days==3  5 to 6 days==4  7 days==5 | **T18** |
| During the past 7 days, on how many days has anyone smoked in your presence, at any outdoor public place such as a playground, sidewalk, entrances to buildings, parks or beaches?  0 days==1  1 to 2 days==2  3 to 4 days==3  5 to 6 days==4  7 days==5 | **T19** | During the past 30 days, did you see anyone smoke inside the school building or outside on school property?  Yes==1  No==2  Not in school==3 | **T20** |
| Do you think the smoke from other people’s tobacco smoking is harmful to you?  Definitely not==1  Probably not==2  Probably yes==3  Definitely yes==4 | **T21** | The last time you **smoked** cigarettes during the past 30 days, how did you get them?  I did not smoke any cigarettes during the past 30 days==1  I bought them in a store or shop==2  I bought them from a street vendor==3  I bought them at a kiosk==4  I got them from someone else==5 | **T22** |
| During the past 30 days, did anyone refuse to sell you cigarettes because of your age?  I did not try to buy cigarettes during the past 30 days==1  Yes, someone refused to sell me cigarettes because of my age==2  No, my age did not keep me from buying cigarettes==3 | **T23** | The last time you **bought** cigarettes during the past 30 days, how did you get them?  I did not buy cigarettes during the past 30 days==1  I bought them in a pack==2  I bought individual sticks/ singles==3  I bought them in a carton==4  I bought them in rolls==5  I bought tobacco and rolled my own==6 | **T24** |
| During the past 30 days, did you see or hear any anti-tobacco media messages on TV, radio, internet, billboards, posters, newspapers, magazines or movies?  Yes==1  No==2 | **T25** | During the past 30 days, did you see or hear any anti-tobacco messages at sports events, concerts or community events or social gatherings?  I did not go to any such events==1  Yes==2  No==3 | **T26** |
| During the past 30 days, did you see any health warnings on cigarette packages?  Yes, but I didn’t think much of them==1  Yes, and they led me to think about quitting smoking or not starting smoking==2  No==3 | **T27** | During the past 12 months, were you taught in any of your classes about the dangers of tobacco use?  Yes==1  No==2  I don’t know==3  Not in school==4 | **T28** |
| During the past 30 days, did you see any people using tobacco on TV, in videos, or in movies?  I did not watch TV, video or movies==1  Yes==1  No==2 | **T29** | During the past 30 days, did you see any advertisements or promotions for tobacco products at points of sale such as supermarkets, kiosks, malls, shops?  I did not visit any points of sale==1  Yes==2  No==3 | **T30** |
| Would you ever use or wear something that has a tobacco company or tobacco product name or picture on it such as a t-shirt, hat, or sunglasses?  Yes==1  Maybe==2  No==3 | **T31** | Do you have something for example a t-shirt, pen, backpack with a tobacco product brand logo on it?  Yes==1  No==2 | **T32** |
| Has a person working for a tobacco company ever offered you a free tobacco product?  Yes==1  No==2 | **T33** | If one of your best friends offered you a tobacco product, would you use it?  Definitely not==1  Probably not==2  Probably yes==3  Definitely yes==4 | **T34** |
| At any time during the next 12 months, do you think you will use any form of tobacco?  Definitely not==1  Probably not==2  Probably yes==3  Definitely yes==4 | **T35** | Once someone has started smoking tobacco, do you think it would be difficult for them to quit?  Definitely not==1  Probably not==2  Probably yes==3  Definitely yes==4 | **T36** |
| Do you think smoking tobacco helps people feel more comfortable or less comfortable at celebrations, parties or in other social gatherings?  More comfortable==1  Less comfortable==2  No difference whether smoking or not==3 | **T37** | Do agree or disagree with the following “I think I might enjoy smoking a cigarette.”  I currently smoke cigarettes==1  Strongly agree==2  Agree==3  Disagree==4  Strongly disagree==5 | **T38** |
| How difficult do you think it would be for you to avoid smoking and using tobacco?  I do not smoke or use tobacco==1  Impossible==2  Very difficult==3  Fairly difficult==4  Fairly easy==5  Very easy==6  I do not know==7 | **T39** | On a scale of 0-100, can you rate how certain you are that you can avoid smoking and using tobacco where  0-----Cannot at all  50-----Can do moderately  100-----Highly certain can do  Rate___________ | **T40** |
| Do you think it would be beneficial to your health for you to avoid smoking and using tobacco?  Yes==1  No==2  If yes, how beneficial to your health would it be for you to avoid smoking and using tobacco?  Very beneficial==1  Moderately beneficial==2  A little beneficial==3 | **T41a**  **T41b** |  |  |

**Section 5: Sanitation and Hygiene**

| **Question** | **Code** | **Question** | **Code** |
| --- | --- | --- | --- |
| Is there a source of clean water for drinking at school?  Yes==1  No==2  Not in school==3 | **H1** | During the past 30 days, how did you usually wash your hands before eating?  I did not wash my hands before eating during the past 30 days==1  In a dish of water used by others==2  In a dish of water used only by me==3  Under running water==4  Some other way==5 | **H2** |
| During the past 30 days, how did you usually wash your hands before eating at school?  I did not wash my hands before eating during the past 30 days==1  In a dish of water used by others==2  In a dish of water used only by me==3  Under running water==4  Some other way==5  Not in school==6 | **H3** | During the past 30 days, how often did you wash your hands before eating at school?  Never==1  Rarely==2  Sometimes==3  Most of the time==4  Always==5  Not in school==6 | **H4** |
| During the past 30 days, how often did you wash your hands after using the toilet or latrine at school?  Never==1  Rarely==2  Sometimes==3  Most of the time==4  Always==5  Not in school==6 | **H6** | During the past 30 days, how often did you use soap when washing your hands at school?  I did not wash my hands at school==1  Never==2  Rarely==3  Sometimes==4  Most of the time==5  Always==6  Not in school==7 | **H7** |
| Is there a place for you to wash your hands after using the toilet or latrine at school?  There are not toilets or latrines at school==1  Yes==2  No==3  Not in school==4 | **H8** | Is there a place for you to wash your hands before eating at school?  Yes==1  No==2  Not in school==3 | **H9** |
| Do you bring water from home to drink while you are at school?  Yes==1  No==2  Not in school==3 | **H10** | How often do you drink water from the water source at school?  There is not a water source at school==1  Never==2  Rarely==3  Sometimes==4  Most of the time==5  Always==6  Not in school==7 | **H11** |
| During the past 30 days, how often did you use the toilets or latrines at school?  There are no toilets or latrines at school==1  Never==2  Rarely==3  Sometimes==4  Most of the time==5  Always==6  Not in school==7 | **H12** | Are there separate toilets or latrines for boys and girls at school?  There are no toilets or latrines at school==1  Yes==2  No==3  Not in school==4 | **H13** |
| Are the toilets or latrines safe at school?  There are no toilets or latrines at school==1  Yes==2  No==3  Not in school==4 | **H14** | Are the toilets or latrines clean at school?  There are no toilets or latrines at school==1  Yes==2  No==3  Not in school==4 | **H15** |
| Are the toilets or latrines easy to get to at school?  There are no toilets or latrines at school==1  Yes==2  No==3  Not in school==4 | **H16** | Are the toilets or latrines private at school  There are no toilets or latrines at school==1  Yes==2  No==3  Not in school==4 | **H17** |
| **Oral Health** | | | |
| How would you describe the health of your teeth?  Excellent==1  Very good==2  Good==3  Average==4  Poor==5  Very poor==6 | **H18** | How would you describe the health of your gums?  Excellent==1  Very good==2  Good==3  Average==4  Poor==5  Very poor==6 | **H19** |
| During the past 12 months, did a tooth ache cause you to miss classes at school  Yes==1  No==2 | **H20** | During the past 12 months, how often did you have a tooth ache or feel discomfort because of your teeth?  Never==1  Rarely==2  Sometimes==3  Most of the time==4  Always==5 | **H21** |
| Do you use toothpaste that contains fluoride?  Yes==1  No==2  I do not know==3 | **H22** | When was the last time you saw a dentist for a checkup, exam, teeth cleaning or other dental work?  During the past 12 months==1  Between 12 and 24 months ago==2  More than 24 months ago==3  Never==4  I do not know==5 | **H23** |
| During the past 12 months, how many times did you go to the dentist?  0 times==1  1 time==2  2 times==3  3 times==4  4 times==5  5 or more times==6 | **H24** | What was the main reason for your last visit to the dentist?  I have never been to the dentist==1  Something was wrong with my teeth or gums==2  For follow-up treatment from an earlier visit==3  For a check-up or exam==4  I do not know==5 | **H25** |
| Which of the following do you most often use to clean your teeth or gums?  Toothbrush==1  Wooden toothpicks==2  Plastic toothpicks==3  Dental floss or thread==4  Charcoal==5  Chew stick==6  Something else==7 | **H26** | Do you have difficulty biting hard foods?  Yes==1  No==2 | **H27** |
| Are you satisfied with the appearance of your teeth?  Yes==1  No==2 | **H28** | Do you have difficulty chewing?  Yes==1  No==2 | **H29** |
| Do you avoid smiling or laughing because of how your teeth look  Yes==1  No==2 | **H30** | Do other students in your school make fun of your teeth?  Yes==1  No==2 | **H31** |
| How many cavities have you had in your permanent teeth?  0 cavities==1  1 cavity==2  2 or 3 cavities==3  4 or 5 cavities==4  6 or more cavities==5  I do not know==6 | **H32** |  |  |
| **Knowledge, Skills, and Sources of Information** | | | |
| During this school year, were you taught in any of your classes the importance of hand washing?  Yes==1  No==2  I do not know==3  Not in school==4 | **H33** | During this school year, were you taught in any of your classes the importance cleaning or brushing your teeth?  Yes==1  No==2  I do not know==3  Not in school==4 | **H34** |
| During this school year, were you taught in any of your classes where to get treatment for worms (de-worming)?  Yes==1  No==2  I do not know==3  Not in school==4 | **H35** | During this school year, were you taught in any of your classes how to avoid infection with worms?  Yes==1  No==2  I do not know==3  Not in school==4 | **H36** |

**Section 6: Mental Health**

| **Question** | **Code** | **Question** | **Code** |
| --- | --- | --- | --- |
| During the past 12 months, how often have you been so worried about something that you wanted to use alcohol or other drugs to feel better?  Never==1  Rarely==2  Sometimes==3  Most of the time==4  Always==5 | **M1** | During the past 12 months, how often have you been so worried about something that you could not eat or did not feel hungry?  Never==1  Rarely==2  Sometimes==3  Most of the time==4  Always==5 | **M2** |
| During the past 12 months, how often have you had a hard time focusing on your homework or other things you had to do?  Never==1  Rarely==2  Sometimes==3  Most of the time==4  Always==5 | **M3** | Have you attempted suicide during the past 12 months?  Yes==1  No==2 | **M4** |
| If you attempted suicide during the past 12 months, did any attempt result in any injury, poisoning, or overdose that had to be treated by a doctor or nurse?  Yes==1  No==2 | **M5** |  |  |
| **Knowledge, Attitudes, Skills and Sources of Information** | | | |
| During this school year, were you taught in any of your classes how to manage anger?  Yes==1  No==2 I do not know==3  Not in school==4 | **M6** | During this school year, were you taught in any of your classes signs of depression and suicidal behavior?  Yes==1  No==2 I do not know==3  Not in school==4 | **M7** |
| During this school year, were you taught in any of your classes what to do if a friend is thinking about suicide?  Yes==1  No==2 I do not know==3  Not in school==4 | **M8** | During this school year, were you taught in any of your classes how to handle stress in healthy ways?  Yes==1  No==2 I do not know==3  Not in school==4 | **M9** |

**Section 7: Physical Activity**

| **Question** | **Code** | **Question** | **Code** |
| --- | --- | --- | --- |
| During the past 12 months, on how many sports teams did you play?  0 teams==1  1 team==2  2 teams==3  3 or more teams==4 | **P1** | During the past 7 days, on how many days did you do exercises to strengthen or tone your muscles, such as push-ups, sit-ups, or weight lifting?  0 days==0  1 day==1  2 days==2  3 days==3  4 days==4  5 days==5  6 days==6  7 days==7 | **P2** |
| During the past 7 days, on how many days did you do stretching exercises such as toe touching, knee bending, or leg stretching?  0 days==0  1 day==1  2 days==2  3 days==3  4 days==4  5 days==5  6 days==6  7 days==7 | **P3** | On an average school night, how many hours of sleep do you get?  4 or less hours==1  5 hours==2  6 hours==3  7 hours==4  8 hours==5  9 hours==6  10 or more hours==7  Not in school==4 | **P4** |
| Do you do any work that involves vigorous-intensity activities that causes large increases in breathing or heart rate like sweeping/raking the compound, brisk walking upstairs, fast cycling like boda boda bicycle, carrying heavy load >20kg for at least 10 minutes continuously?  Yes==1  No==2 | **P5** | In a typical week, on how many days do you do vigorous-intensity activities?  _____days | **P6** |
| How much time do you spend doing vigorous-intensity activities on a typical day?  _____hours  _____minutes | **P7**  **P7a**  **P7b** | Do you do any work that involves moderate-intensity activity that causes small increases in breathing or heart rate such as brisk walking, digging, housework/ domestic chores, building tasks, carrying food items or cooking or carrying heavy load <20kg for at least 10 minutes continuously?  Yes==1  No==2 | **P8** |
| In a typical week, on how many days do you do moderate-intensity activities?  _____days | **P9** | How much time do you spend doing moderate-intensity activities on a typical day?  _____hours  _____minutes | **P10**  **P10a**  **P10b** |
| Do you walk or use a bicycle for at least 10 minutes continuously to get to and from places?  Yes==1  No==2 | **P11** | In a typical week, on how many days do you walk or bicycle for at least 10 minutes continuously to get to and from places?  _____days | **P12** |
| How much time do you spend walking or bicycling for travel on a typical day?  _____hours  _____minutes | **P13**  **P13a**  **P13b** | Do you do any vigorous-intensity sports, fitness or recreational activities that cause large increases in breathing or heart rate like running, fast cycling, fast swimming, netball, basketball, football, rugby etc for at least 10 minutes continuously?  Yes==1  No==2 | **P14** |
| In a typical week, on how many days do you do vigorous-intensity sports, fitness or recreational activities?  _____days | **P15** | How much time do you spend doing vigorous-intensity sports, fitness or recreational activities on a typical day?  _____hours  _____minutes | **P16**  **P16a**  **P16b** |
| Do you do any moderate-intensity sports, fitness or recreational activities that cause a small increase in breathing or heart rate like brisk walking, cycling, swimming, volleyball, gardening etc for at least 10 minutes continuously?  Yes==1  No==2 | **P17** | In a typical week, on how many days do you do moderate-intensity sports, fitness or recreational activities?  _____days | **P18** |
| How much time do you spend doing moderate-intensity sports, fitness or recreational activities on a typical day?  _____hours  _____minutes | **P19**  **P19a**  **P19b** | How much time do you usually spend sitting or reclining on a typical day?  _____hours  _____minutes | **P20**  **P20a**  **P20b** |
| How much time do you spend on social media each day?  _____hours  _____minutes | **P21** |  |  |
| **Knowledge, Attitudes, Skills and Sources of Information** | | | |
| During this school year, were you taught in any of your classes how to develop a physical fitness plan for yourself?  Yes==1  No==2 I do not know==3  Not in school==4 | **P22** | During this school year, were you taught in any of your classes about preventing injury during physical activity?  Yes==1  No==2 I do not know==3  Not in school==4 | **P23** |
| During this school year, were you taught in any of your classes the benefits of physical activity?  Yes==1  No==2 I do not know==3  Not in school==4 | **P24** | During this school year, were you taught in any of your classes about opportunities for physical activity in your community?  Yes==1  No==2 I do not know==3  Not in school==4 | **P25** |
| How difficult do you think it would be for you to engage in at least 60 minutes of moderate to vigorous-intensity physical activity daily [5-17years] OR 150 minutes of moderate-intensity activity in a week or at least 75 minutes of vigorous-intensity activity in a week [18-24years] ?  I do not engage in any physical activity==1  Impossible==2  Very difficult==3  Fairly difficult==4  Fairly easy==5  Very easy==6  I do not know==7 | **P26** | On a scale of 0-100, can you rate how certain you are that you can engage in at least 60 minutes of moderate to vigorous-intensity physical activity daily [5-17years] OR 150 minutes of moderate-intensity activity in a week or at least 75 minutes of vigorous-intensity activity in a week [18-24years] where  0-----Cannot at all  50-----Can do moderately  100-----Highly certain can do  Rate___________ | **P27** |
| Do you think it would be beneficial to your health for you to engage in at least 60 minutes of moderate to vigorous-intensity physical activity daily [5-17years] OR 150 minutes of moderate-intensity activity in a week or at least 75 minutes of vigorous-intensity activity in a week [18-24years]?  Yes==1  No==2  If yes, how beneficial to your health would it be for you to engage in at least 60 minutes of moderate to vigorous-intensity physical activity daily [5-17years] OR 150 minutes of moderate-intensity activity in a week or at least 75 minutes of vigorous-intensity activity in a week [18-24years]?  Very beneficial==1  Moderately beneficial==2  A little beneficial==3 | **P28a**  **P28b** | Have you ever received advice from a health practitioner, friend, relative to engage in physical activity?  Yes==1  No==2 | **P29** |

**Section 8: Protective factors**

| **Question** | **Code** | **Question** | **Code** |
| --- | --- | --- | --- |
| Do you think of yourself as a religious or spiritual person?  Yes==1  No==2 | **R1** |  |  |
| **Connection to parents** | | | |
| During the past 30 days, how often did your parents or guardians comfort you?  Never==1  Rarely==2  Sometimes==3  Most of the time==4  Always==5 | **R2** | During the past 30 days, how often did your parents or guardians understand you?  Never==1  Rarely==2  Sometimes==3  Most of the time==4  Always==5 | **R3** |
| During the past 30 days, how often did your parents or guardians give you attention and listen to you?  Never==1  Rarely==2  Sometimes==3  Most of the time==4  Always==5 | **R4** | During the past 30 days, how often did your parents or guardians have open communication with you?  Never==1  Rarely==2  Sometimes==3  Most of the time==4  Always==5 | **R5** |
| During the past 30 days, how often did your parents or guardians show you affection?  Never==1  Rarely==2  Sometimes==3  Most of the time==4  Always==5 | **R6** | During the past 30 days, how often did your parents or guardians spend time with you?  Never==1  Rarely==2  Sometimes==3  Most of the time==4  Always==5 | **R7** |
| During the past 30 days, how often did your parents or guardians praise you?  Never==1  Rarely==2  Sometimes==3  Most of the time==4  Always==5 | **R8** | During the past 30 days, how often did your parents or guardians give you advice and guidance?  Never==1  Rarely==2  Sometimes==3  Most of the time==4  Always==5 | **R9** |
| During the past 30 days, how often did your parents or guardians give you money?  Never==1  Rarely==2  Sometimes==3  Most of the time==4  Always==5 | **R10** |  |  |
| **Parental Disrespect of Individuality/ Worth** | | | |
| During the past 30 days, how often did your parents or guardians ridicule you or put you down (for example say you were stupid or useless)?  Never==1  Rarely==2  Sometimes==3  Most of the time==4  Always==5 | **R11** | During the past 30 days, how often did your parents or guardians expect too much of you (for example, to do better in school or be a better person)?  Never==1  Rarely==2  Sometimes==3  Most of the time==4  Always==5 | **R12** |
| During the past 30 days, how often did your parents or guardians embarrass you in public or in front of your friends?  Never==1  Rarely==2  Sometimes==3  Most of the time==4  Always==5 | **R13** | During the past 30 days, how often did your parents or guardians unfairly compare you to someone else (such as to your brother or sister or to themselves)?  Never==1  Rarely==2  Sometimes==3  Most of the time==4  Always==5 | **R14** |
| During the past 30 days, how often did your parents or guardians not respect you as a person (for example, not let you talk or favor someone else more than you)?  Never==1  Rarely==2  Sometimes==3  Most of the time==4  Always==5 | **R15** | During the past 30 days, how often did your parents or guardians ignore you (for example walk away from you or not pay attention to you)?  Never==1  Rarely==2  Sometimes==3  Most of the time==4  Always==5 | **R16** |
| During the past 30 days, how often did your parents or guardians try to make you feel guilty for something you had done or something they thought you should do?  Never==1  Rarely==2  Sometimes==3  Most of the time==4  Always==5 | **R17** |  |  |
| **Parent Monitoring and Knowledge of Friends and Activities** | | | |
| During the past 30 days, how often did your parents or guardians try to know who your friends were?  Never==1  Rarely==2  Sometimes==3  Most of the time==4  Always==5 | **R18** | During the past 30 days, how often did your parents or guardians really know who your friends were?  Never==1  Rarely==2  Sometimes==3  Most of the time==4  Always==5 | **R19** |
| During the past 30 days, how often did your parents or guardians try to know where you went at night?  Never==1  Rarely==2  Sometimes==3  Most of the time==4  Always==5 | **R20** | During the past 30 days, how often did your parents or guardians really know where you went at night?  Never==1  Rarely==2  Sometimes==3  Most of the time==4  Always==5 | **R21** |
| During the past 30 days, how often did your parents or guardians try to know how you spent your money?  Never==1  Rarely==2  Sometimes==3  Most of the time==4  Always==5 | **R22** | During the past 30 days, how often did your parents or guardians really know how you spent your money?  Never==1  Rarely==2  Sometimes==3  Most of the time==4  Always==5 | **R23** |
| During the past 30 days, how often did your parents or guardians try to know where you were most afternoons after school?  Never==1  Rarely==2  Sometimes==3  Most of the time==4  Always==5 | **R24** | During the past 30 days, how often did your parents or guardians really know where you were most afternoons after school?  Never==1  Rarely==2  Sometimes==3  Most of the time==4  Always==5 | **R25** |
| During the past 30 days, how often did your parents or guardians try to know what you did with your free time?  Never==1  Rarely==2  Sometimes==3  Most of the time==4  Always==5 | **R26** |  |  |
